# Redescription of the enigmatic neotropical inquiline *Paramyrmetes foveipennis* Bruch, 1929 with notes on myrmecophily (Coleoptera, Histeridae) in the Saprininae subfamily

**DOI:** 10.3897/zookeys.675.12690

**Published:** 2017-05-19

**Authors:** Tomáš Lackner

**Affiliations:** 1 Bavarian State Collection of Zoology, Münchhausenstraße 21, 81247 Munich, Germany

**Keywords:** Coleoptera, Histeridae, myrmecophily, *Paramyrmetes*, Saprininae

## Abstract

The poorly-known and highly autapomorphic myrmecophilous Neotropical taxon *Paramyrmetes foveipennis* Bruch, 1929 is redescribed, figured and its lectotype designated. Notes on the evolution of the inquilinous lifestyle (myrmecophily) in the subfamily Saprininae are given.

## Introduction

Several years ago the first higher-level phylogeny of the Saprininae was published, where all but three genera and subgenera of this subfamily were included (Lackner 2014). The outstanding taxa were Auchmosaprinus Wenzel, 1962, a subgenus of Xerosaprinus Wenzel, 1962; *Satrapister* Bickhardt, 1912; and *Paramyrmetes* Bruch, 1929. *Satrapister* was the subject of a separate paper (Lackner 2016), whereas *Paramyrmetes* is the subject of the present study. The monotypic genus *Paramyrmetes* was described by Carlos Bruch based on five specimens collected by himself and Prof. J. Hubrich in 1923 and 1927, respectively, inside refuse chambers of nests of the ant Pogonomyrmex cunicularis var. carnivora Santschi, 1925 (= currently a junior synonym of *Pogonomyrmex serpens* Santchi, 1922) in the Santa Fé province of Argentina (Bruch 1929). Bruch had difficulties with the systematic placement of this species, and, referring to the key to histerid genera available to him at the time (Bickhardt 1916), placed the taxon into the Saprininae subfamily between ant-inquilines *Myrmetes* Marseul, 1862 and *Platysaprinus* Bickhardt, 1916. The same author noted the peculiarities of *Paramyrmetes*: the rounded, tongue-like labrum and rectangular head, with a broad protruding clypeus that is fused with the frons, and the absent frontal stria. The position of the antennae placed pressed against and parallel to the prosternal process when the head is retracted as well as the prosternum itself resembles, according to Bruch, the taxon *Platysaprinus* (currently a subgenus of *Euspilotus* Lewis, 1907). Bruch also noted several autapomorphies of this genus: the presence of elytral depressions as well as reduced dorsal elytral striae. He pointed out the structure of the antennae and the dilated tibiae as putative morphological adaptations to myrmecophily and observed that *Paramyrmetes foveipennis* was the first recorded (beetle) ant-guest of Pogonomyrmex cunicularis var. carnivora (= *Pogonomyrmex serpens* Santchi, 1922). In his catalogues (1984, 1997, 2011) Mazur consistently placed *Paramyrmetes* between the Australian *Tomogenius* Marseul, 1862 and Palaearctic *Myrmetes* Marseul, 1862.

In this paper, *Paramyrmetes* is re-described based on the type material. Habitus images as well as drawings of male genitalia are provided. This work represents another contribution to the systematics and higher taxonomy of the Saprininae (see e.g. Lackner 2014 or 2016 and the references therein).

## Material and methods

A dry-mounted syntype of *Paramyrmetes foveipennis* was relaxed in warm water for several hours. After removal from the original card, it was side-mounted on a triangular point and examined under a Nikon 102 binocular microscope and viewed with diffuse light. Male genitalia were first macerated in 10% KOH solution for about 3 hours, cleared in 80% alcohol and macerated in lactic acid with fuchsine, incubated at 60 ºC for another 30 minutes, and subsequently cleared in 80% alcohol and then observed in α-terpineol in a small dish. Digital photographs of male genitalia were taken by a Nikon 4500 Coolpix camera and edited in Adobe Photoshop CS5. Genitalia drawings based on the photographs, or direct observations were produced with the aid of Hakuba klv-7000 light box. Habitus photographs were taken by F. Slamka (Bratislava, Slovakia). The specimen was measured with an ocular micrometer. Beetle terminology follows that of Ôhara (1994) and Lackner (2010).

The specimen examined for this study is deposited in the following collection:

**MNNC** Museo Nacional de Historia Natural (Santiago de Chile, Chile).

**Abbreviations.** Abbreviations of morphological measurements follow Ôhara (1994) and are used throughout the text as follows:

**APW** width between anterior angles of pronotum

**EL** length of elytron along elytral suture

**EW** maximum width between outer margins of elytra

**PEL** length between anterior angles of pronotum and apices of elytra

**PPW** width between posterior angles of pronotum.

## Results

### Paramyrmetes

Taxon classificationAnimaliaColeopteraHisteridae

Bruch, 1929

Paramyrmetes Bruch, 1929: 421. Type species Paramyrmetes foveipennis Bruch, 1929: 422, by monotypy.
Paramyrmetes : Mazur (1984): 107; Mazur (1997): 217; Mazur (2011): 178.

#### Diagnosis.

Medium-sized reddish-brown shining asetose Saprininae beetle with completely punctate and shagreened dorsal cuticle, broadly rectangular head; frontal and supraorbital striae absent, labrum tongue-shaped. Dorsal elytral striae strongly reduced; apical third of elytra deeply depressed; metaventrite and first visible abdominal ventrite with (striolate) depression. Pygidium with prominent round ornamentation; tibiae dilated.

#### Differential diagnosis.

Based on the autapomorphies outlined above, *Paramyrmetes* cannot be confused with any currently known South American Saprininae genus. The overall body coloration, in combination with the depressed apical third of the elytra, metaventral and abdominal depressions and, especially the peculiarly-shaped labrum, will readily set this taxon apart from the other members of the subfamily. Moreover, *Paramyrmetes* possesses pygidial ornamentation in the male sex. Pygidial ornamentation occurs in the Saprininae subfamily rather seldom, and was observed so far only with female specimens of several taxa (e.g. Euspilotus (Neosaprinus) perrisi Marseul, 1872)). According to my knowledge, male pygidial ornamentation has not been reported in the Saprininae subfamily hitherto.

#### Biology.

The type series was found inside the refuse chambers of the ant *Pogonomyrmex serpens* Santchi, 1922. This species is apparently a specialised ant inquiline.

#### Distribution.

Known only from the type series collected in the province of Santa Fé, Argentina (Fig. 17).

#### Remarks.

Although Bruch (1929) mused about the metaventral- and abdominal depressions as a possible sexual character of *Paramyrmetes*, he did not specify the sex of the specimens he examined. As I was only able to examine a single male, I am not sure whether *Paramyrmetes foveipennis* is a sexually dimorphic species or not.

### Paramyrmetes foveipennis

Taxon classificationAnimaliaColeopteraHisteridae

Bruch, 1929

Figs 1 , 2 , 3 , 4 , 5 , 6 , 7 , 8–16 , 17

Paramyrmetes foveipennis Bruch, 1929: 422, figs 1–5, 11; Mazur (1984): 107; Mazur (1997): 217; Mazur (2011): 178.

#### Type material examined.

Lectotype, present designation, ♂, side-mounted on a triangular mounting card, genitalia dismembered, glued to a separate card under the specimen, with the following labels: “Hersilia / “La Geraldina”” (written); followed by: “Prov.S.Fé.II.927 / J. Hubrich” (written); followed by: “con / Pogonomyrmex / v.carnivora” (written); followed by: “Phototypus” (light-green label, written); followed by: “Paramyrmetes / foveipennis / Bruch / C. BRUCH DETERM.” (printed-written); followed by: “ACHADO” (written); followed by: “Paramyrmetes / foveipennis / Bruch, 1929 / LECTOTYPE / Des. T. Lackner 2017” (red label, written) (MNNC). This specimen is undoubtedly one of the syntypes, as Bruch described the species based on a single specimen collected by him and four specimens collected by Prof. Hubrich in Hersilia. The examined specimen belongs to the series collected by Prof. Hubrich. According to Bruch (1929) the type specimens were deposited in his private collection, and the private collections of Reichensperger and Hubrich. It is possible that the private collection of Prof. Hubrich was acquired by MNNC, where the lectotype is housed currently. Lectotype designation fixes the species’ identity; the outstanding four exemplars, whose whereabouts are unknown, would qualify for the paralectotype status.

**Figure 1.**
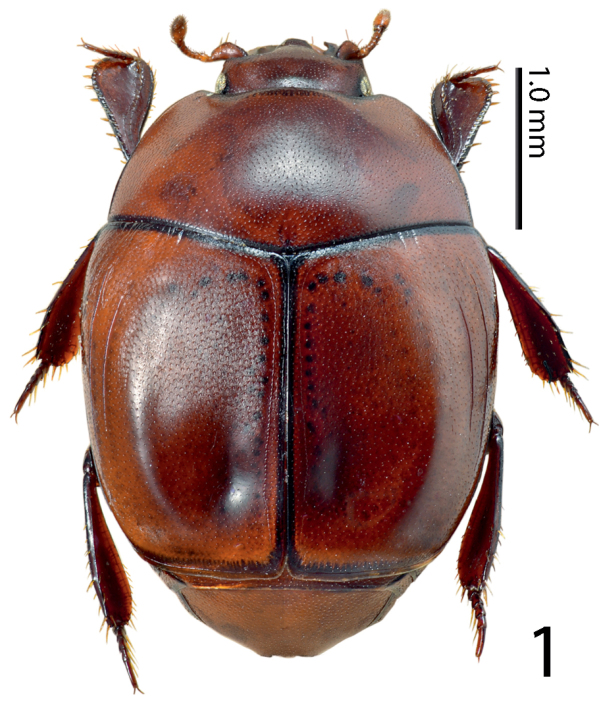
*Paramyrmetes foveipennis* Bruch, 1929, ♂, lectotype, habitus, dorsal view.

**Figure 2.**
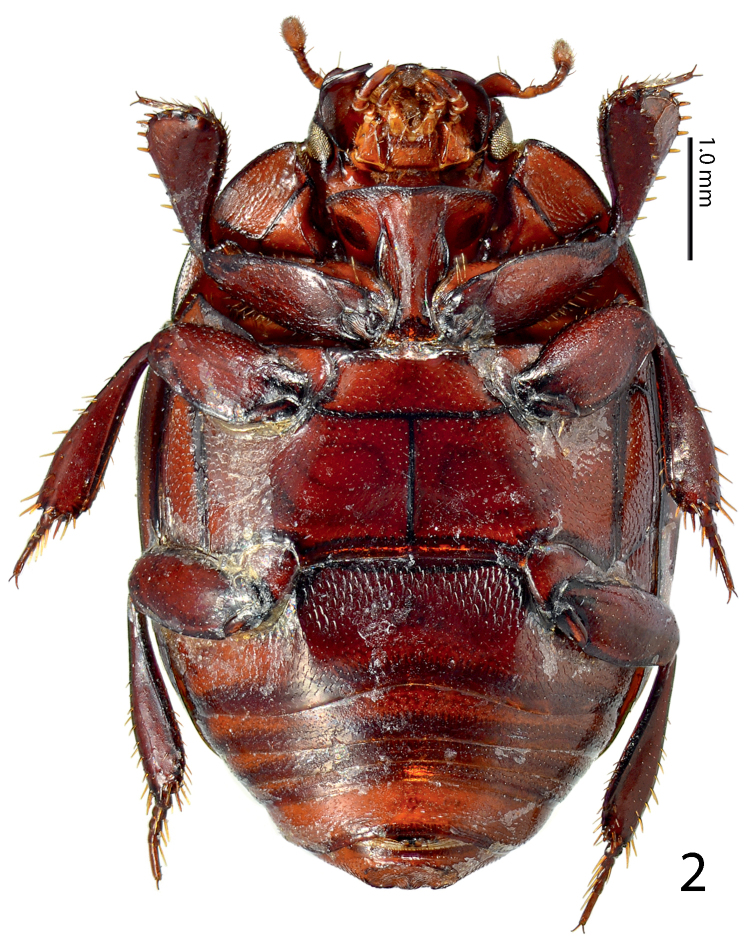
*Paramyrmetes foveipennis* Bruch, 1929, ♂, ditto, ventral view.

#### Re-description.

**Figure 3.**
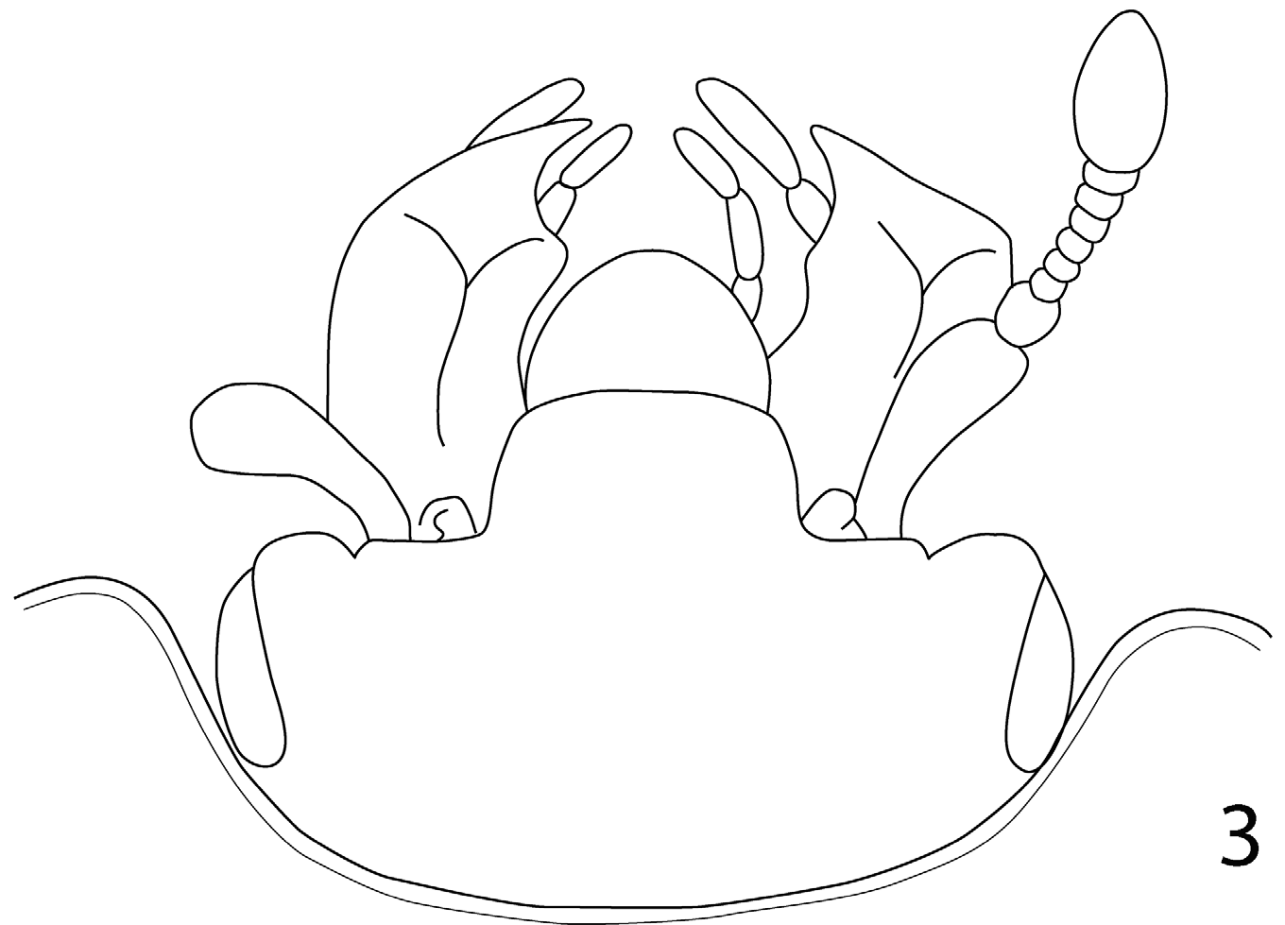
*Paramyrmetes foveipennis* Bruch, 1929, ♂, head, dorsal view, showing the peculiar labrum (redrawn from Bruch 1929).

**Figure 4.**
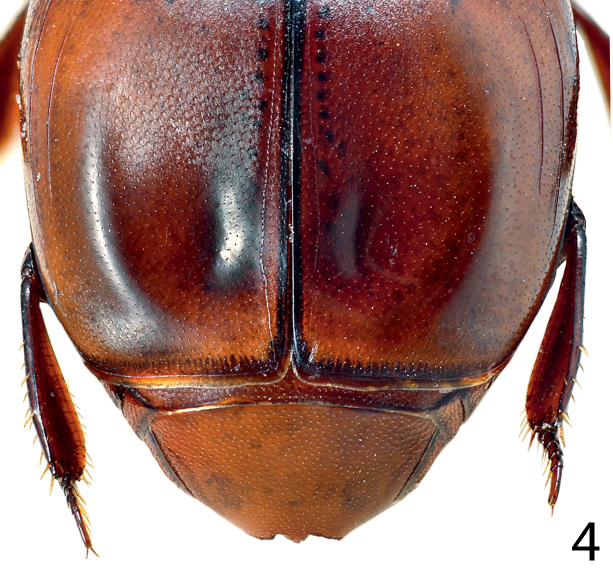
*Paramyrmetes foveipennis* Bruch, 1929, ♂, lectotype, detail of the elytral depression.

**Figure 5.**
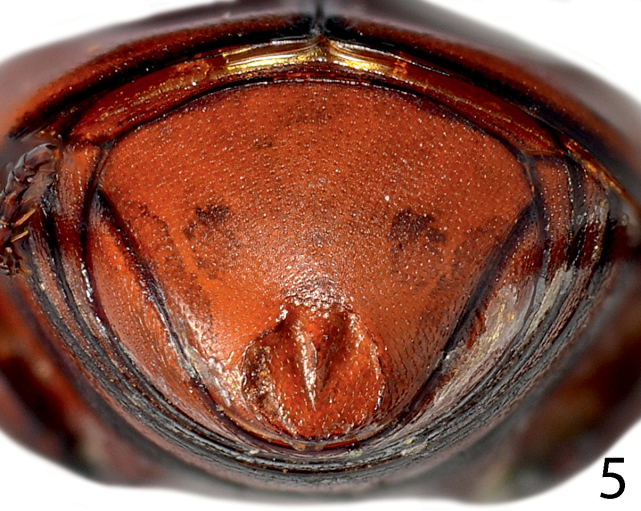
*Paramyrmetes foveipennis* Bruch, 1929, ♂, ditto, pygidium, caudal view.

Body: PEL: 3.00 mm; PPW: 1.00 mm; APW: 2.00 mm; EW: 2.60 mm; EL: 1.70 mm. Body, including appendages reddish-brown (Figs 1–2). Head broad, frons approximately twice as wide as long, with several vague depressions, frontal and supraorbital striae absent; clypeus large, quadrate, sloping down laterally; both frons and clypeus covered with dense fine punctures separated by approximately 1-2 times their diameter, interspaces with alutaceous microsculpture. Eyes flattened, but visible from above; antennal scape slender, with several setae; pedicel approximately as long as following three antennomeres together; 8^th^ antennomere saucer-like. Antennal club oval, on distal half (roughly) with dense microsetae intermingled with several longer setae; sensory structures not examined. Mouthparts: labrum (Fig. 3) unusually large, tongue-shaped; labral pits situated on labral edges, each with two labral setae. Mandibles similar to other taxa of the subfamily, apically pointed, finely punctate. Ultimate palpomere of both labial and maxillary palpi elongate, thin, approximately four times as long as wide. Mentum quadrate, anterior margin with deep semi-circular median emargination; rest of mouthparts not examined. Pronotum broad, approximately twice as wide as long; marginal pronotal stria carinate laterally, weakened behind head. Apical pronotal angles obtuse; pronotal disk with punctation similar to that of head, punctures fine, separated by approximately twice their diameter, interspaces with prominent alutaceous microsculpture. Pronotal hypomeron glabrous. Elytral epipleuron with microscopic punctation, marginal epipleural stria very fine, complete; marginal elytral stria complete, carinate, continued as weakened but complete marginal elytral stria connected to fine, complete sutural elytral stria. Humeral elytral stria fine, doubled, present on basal elytral third approximately; both subhumeral striae lacking. First dorsal elytral stria shortened on approximate basal tenth and apical fifth; second dorsal elytral stria even shorter, shortened on approximate basal fifth and apical third; rest of the elytral striae (apart from fine and complete sutural elytral stria) missing. Elytral disk in anterior two-thirds relatively coarsely, subimbricately punctate, punctures with posterior margins effaced, each with short appressed seta one to two times puncture diameter, interpunctural integument with very dense and prominent alutaceous microsculpture; in posterior third with punctation similar to that of pronotum and near elytral suture with rather deep large circular depression (Fig. 4). Scutellum very small, triangular. Propygidium partly covered by elytra, densely punctate, punctures separated by about their own diameter; pygidium triangular, densely punctate, punctures finer than those of propygidium, separated about their own diameter; apex of pygidium (Fig. 5) with round prominent well-defined depression, depression edges roughly subcarinate, with raised narrow triangular inclusion beginning at basomedial third of margin and ending at apical fifth, inclusion subcarinate in its apical two-thirds.

**Figure 6.**
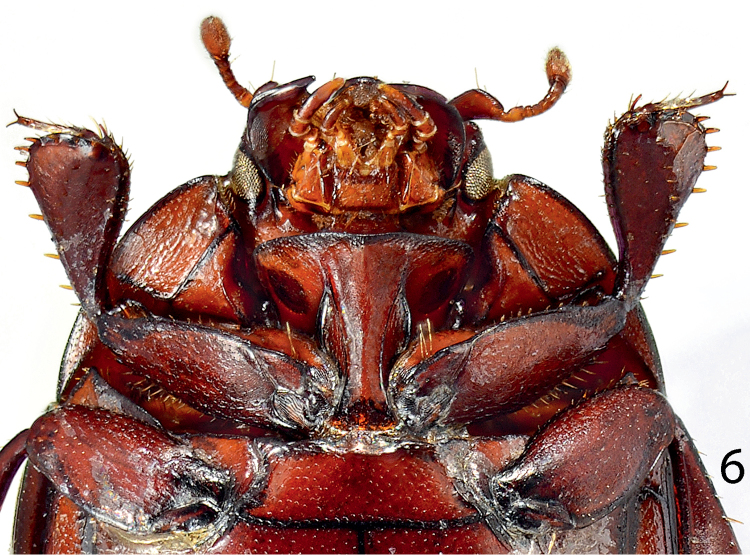
*Paramyrmetes foveipennis* Bruch, 1929, ♂, ditto, prosternum and mesoventrite.

**Figure 7.**
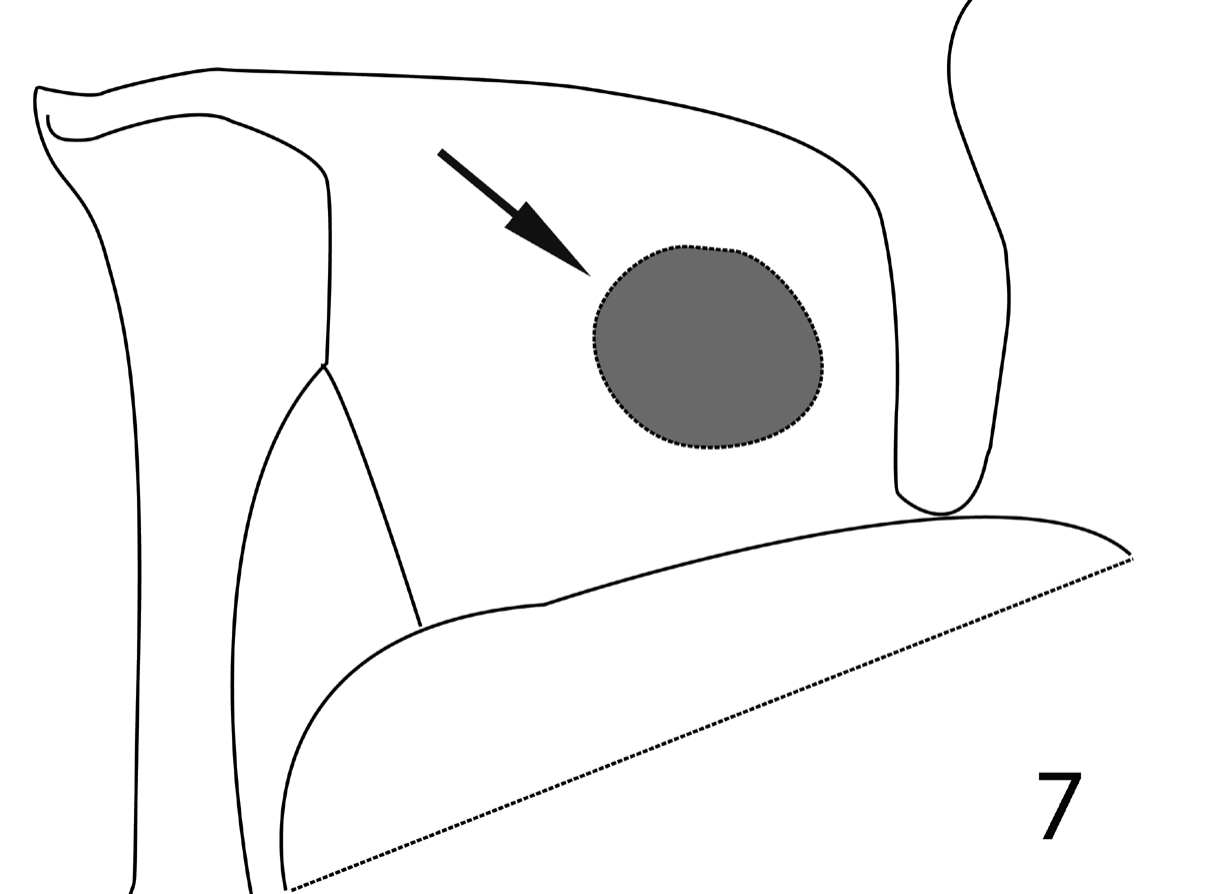
*Paramyrmetes foveipennis* Bruch, 1929, ♂, ditto, schematic detail of the prosternal process showing the curious circle presumed to help with the accommodation of antennal club when head is retracted.

Prosternum: apical margin of prosternum (Fig. 6) rounded, marginal prosternal stria absent; carinal prosternal striae present, largely divergent apically; surface between striae convex. Lateral prosternal striae costate, apically fusing with widely divergent apices of carinal prosternal striae slightly anterior to anterior third; prosternal foveae absent. Prosternal process laterally with a single large dark circular depression presumed to accommodate antennal club when head is retracted (Fig. 7). Mesoventrite: lateral mesoventral stria complete, inwardly arcuate medially. Punctation of mesoventral disk similar to that of pronotum, punctures separated by 1-2 times their diameter, interspaces with microsculpture. Metaventrite: sparsely and finely punctate, lateral metaventral stria absent. Basal half of metaventral disk moderately depressed, this depression continues and occupies entire first visible abdominal ventrite which is strongly striolate (Fig. 2). Lateral disk of metaventrite with subcontiguous large shallow punctures, metepisternum with similar punctation; metepisternal stria thin, complete.

Legs: protibia dilated, outer margin with approximately 14 short denticles, teeth lacking. Protibial spur thin, emerging near tarsal insertion, protarsal groove shallow. Mesotibia and metatibia not particularly dilated, outer margin with sparse denticles.

**Figures 8–16.**
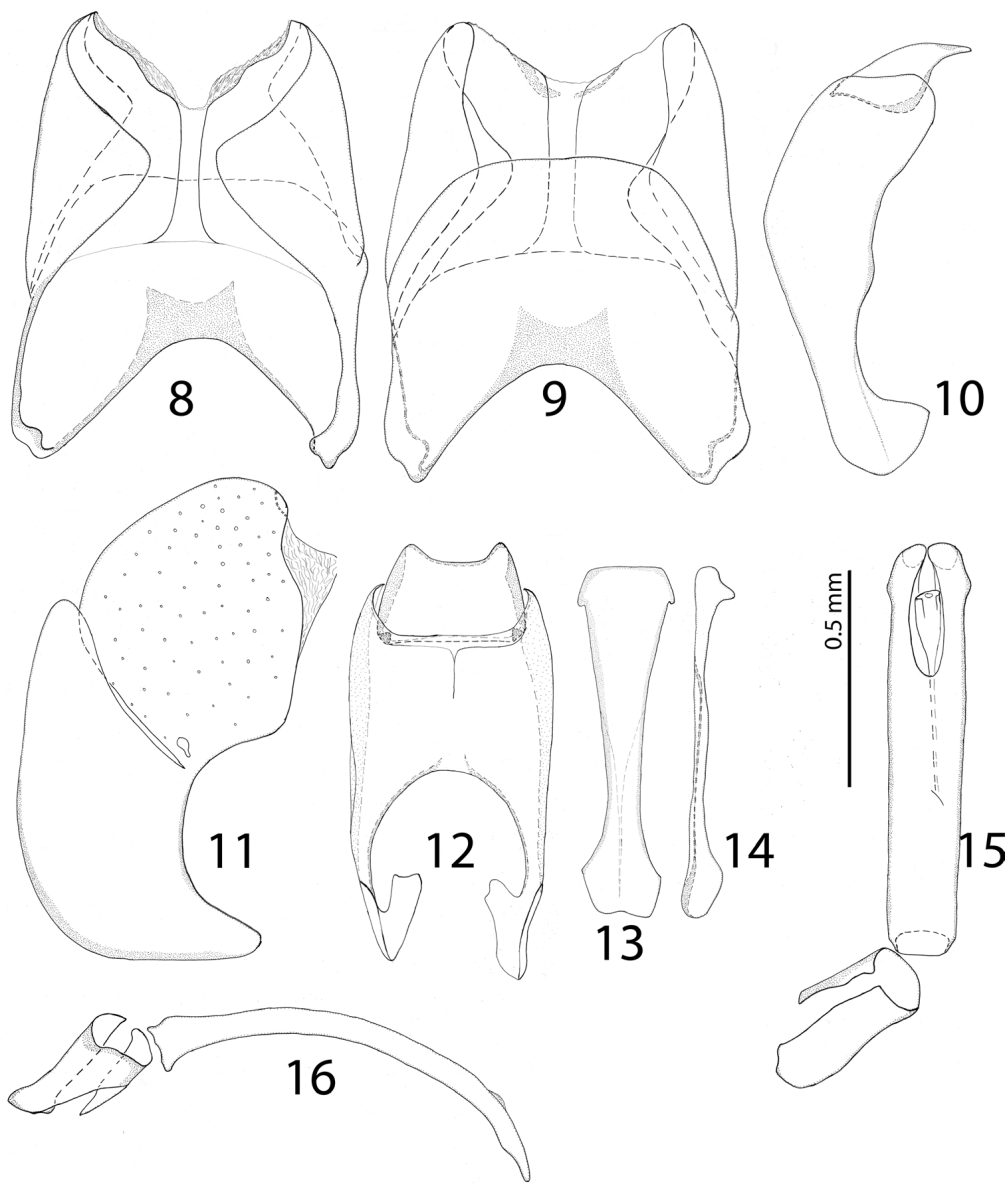
**8** *Paramyrmetes foveipennis* Bruch, 1929, ♂, lectotype, terminalia: eighth sternite and tergite, ventral view **9** ditto, dorsal view **10** ninth and tenth tergites, lateral view **11** eighth sternite and tergite, lateral view **12** ninth and tenth tergites, dorsal view **13** spiculum gastrale (ninth sternite), ventral view **14** ditto, lateral view **15** aedeagus, dorsal view **16** ditto, lateral view.

Male genitalia: 8^th^ sternite (Figs 8–9) apically without setae; 8^th^ tergite (Fig. 9) apically outwardly arcuate; 8^th^ sternite and tergite laterally fused (Fig. 11). Tenth tergite apically inwardly arcuate (Fig. 12); 9^th^ tergite fused dorsally (Fig. 12). Spiculum gastrale (9^th^ sternite) gradually dilated on both ends (Figs 13–14). Aedeagus (Figs 15–16) curved in lateral view, phallobase approximately one fifth of tegmen’s length. Parameres fused on their basal two-thirds. Female unavailable.

**Figure 17.**
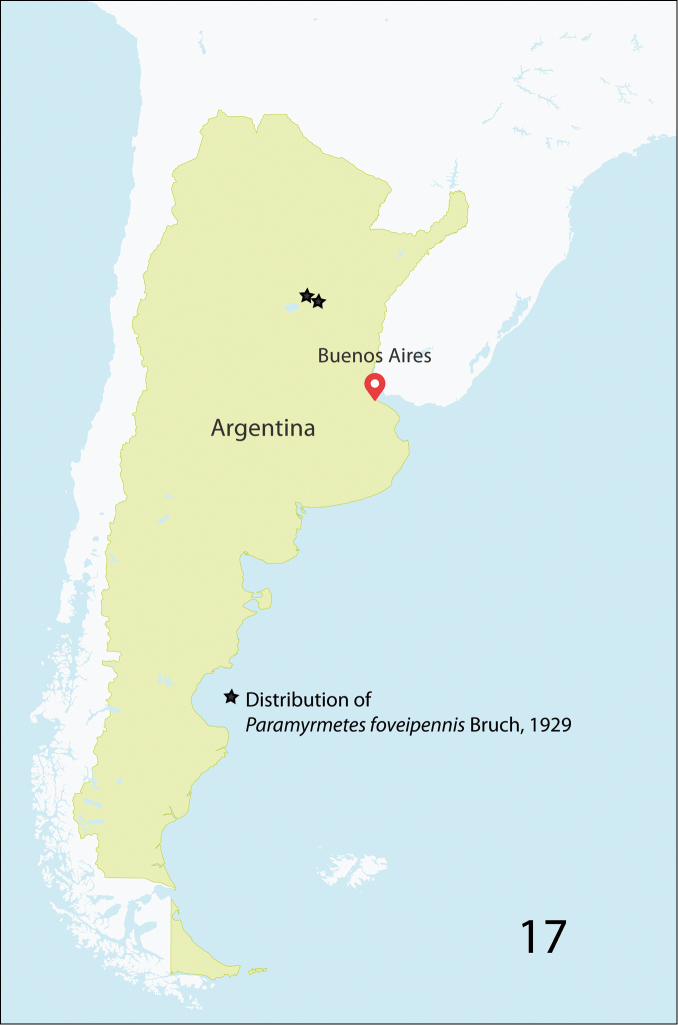
Distribution of *Paramyrmetes foveipennis* Bruch, 1929 in Argentina.

**Figure 18.**
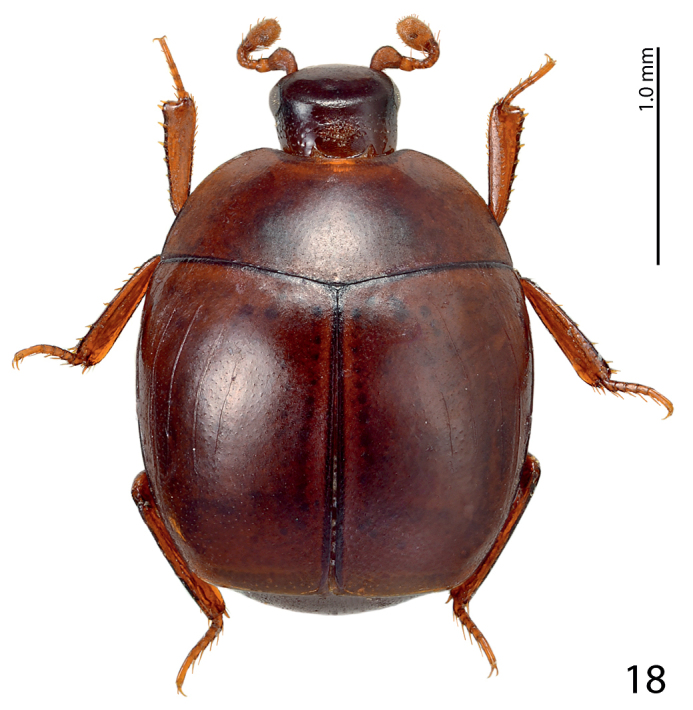
*Myrmetes paykulli* Kanaar, 1979 habitus, dorsal view.

**Figure 19.**
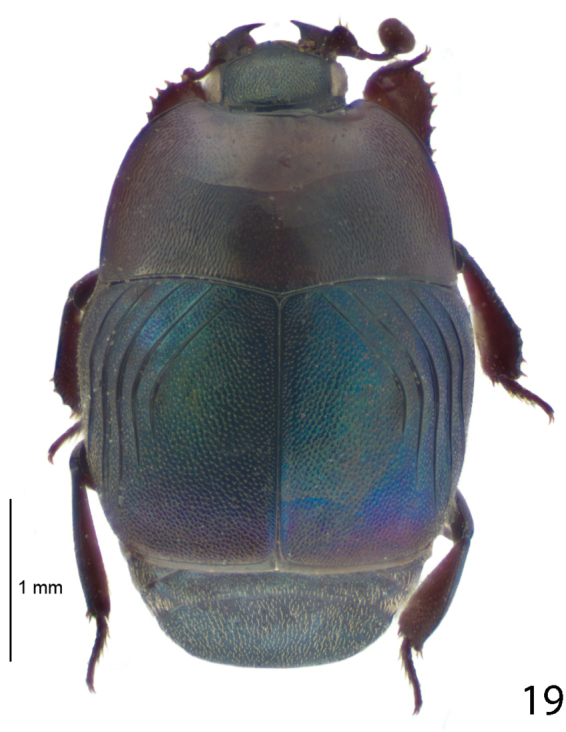
Undescribed genus, Australia habitus, dorsal view.

**Figure 20.**
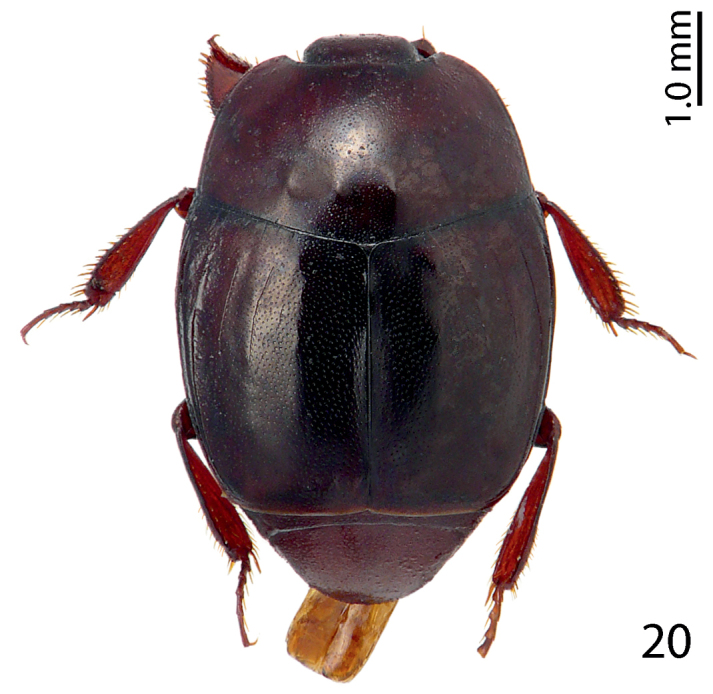
Phoxonotus (Ph.) parvotuberculatus Lackner, 2016 habitus, dorsal view.

**Figure 21.**
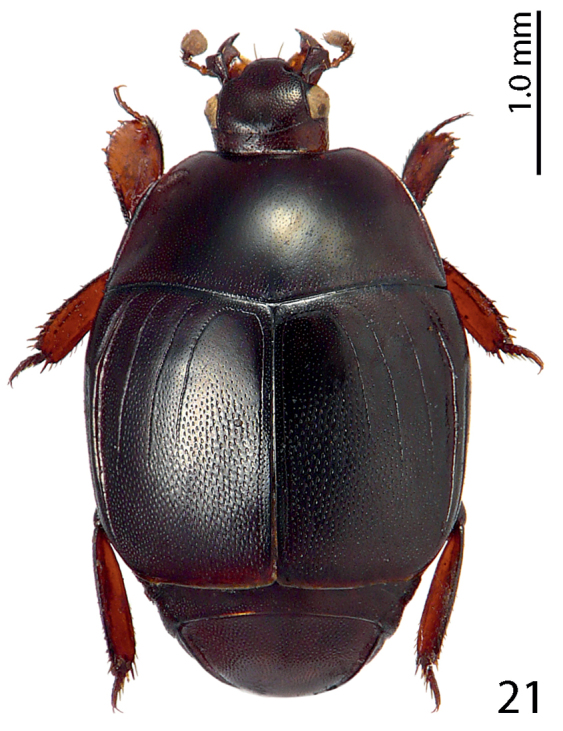
Euspilotus (Platysaprinus) latimanus (Schmidt, 1890) habitus, dorsal view.

**Figure 22.**
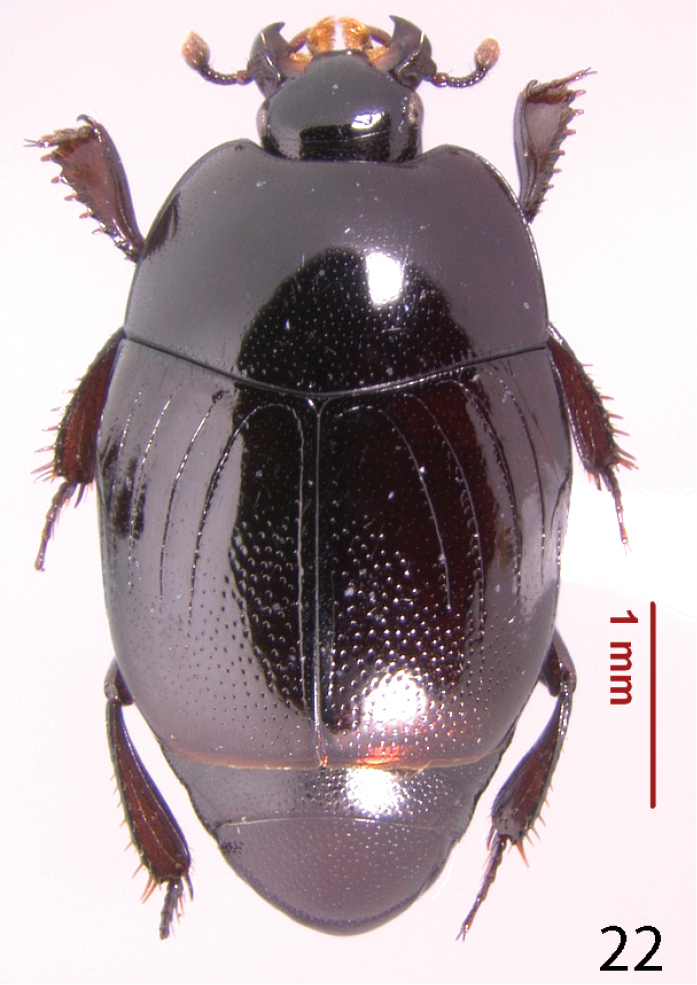
Geomysaprinus (Priscosaprinus) formicus (Hinton, 1935) habitus, dorsal view.

## Discussion

In his catalogues Mazur (1984, 1997, 2011), without any prior analysis, consistently placed *Paramyrmetes* among the more ‘basal’ Saprininae, between the cave-dwelling and inquilinous Australopacific genus *Tomogenius* Marseul, 1862 and Palaearctic ant-inquiline genus *Myrmetes* Marseul, 1862. In my analysis, both *Tomogenius* and *Myrmetes* were found to be near the root of the tree, and, in fact an inquilinous lifestyle of saprinines is inferred to be plesiomorphic for the subfamily (Lackner 2014).

For the present study, I coded the morphological characters of *Paramyrmetes* and included them in the matrix used for my *Satrapister* paper (Lackner 2016). The position of *Paramyrmetes* on the tree was unstable, varying among the taxa near the root (*Myrmetes*, *Erebidus*, *Gnathoncus*, *Tomogenius*, *Microsaprinus*). This instability was mainly due to the impossibility of coding antennal club characters, as the disarticulation of the unique syntype available was not allowed. Furthermore, *Paramyrmetes* exhibits numerous autapomorphies, which are not informative for the phylogenetic reconstruction. Consequently, I decided not to include the new tree here, opting instead to await the results of the molecular analysis (on-going). Despite the unstable position on the tree, *Paramyrmetes*, consistently came out near the root of the tree, in fact corroborating Mazur’s taxon placement. Inclusion of freshly collected specimens into the ongoing molecular analysis would be highly desirable.

Myrmecophily, or an ant-inquilinous lifestyle is rather rare among saprinine histerids and has hitherto been confirmed in six higher taxa with at least three independent evolutionary events leading to it (Lackner 2014; P. Kovarik, pers. communication). It is interesting to note that four out of the six ant-inquiline higher taxa occur in the New World, and three of those are attaphilic (see the table below for details). Several, but not all inquilinous saprinines exhibit peculiar morphological autapomorphies linked to their specialized lifestyles, and these autapomorphies are not similar between the taxa. Among the shared characters could be listed their wholly punctate bodies (except for *Myrmetes*), interrupted to absent frontal stria (all taxa), and occasional lack of sutural elytral stria (present in *Priscosaprinus* and *Paramyrmetes*). The dilated tibiae, which are present in most taxa (exceptions are: *Myrmetes*, *Priscosaprinus* and *Phoxonotus*) probably function to protect larger portion of venter when in repose, since they are able to cover more space. Autapomorphies of the undescribed Australian genus, which include imbricately setose elytra and abdomen (it is hypothesized that these micro-setae function as trichomes secreting appeasing liquid), metepisternal groove for receiving the mesotarsus, and enlarged antennal club are further results of the selection pressures for inquilinous lifestyle. Many of these are also found in other ant-inquiline histerids (e.g. Haeteriinae). The functions of several autapomorphies, e.g. elytral and metepisternal-abdominal depressions, dorsal tubercles, tongue-shaped labrum or pygidial ornamentation is completely unknown. Larvae of ant-inquiline saprinine beetles are unknown and biology generally poorly understood. The table below is presented to outline several putative morphological adaptations presumably linked with ant-inquilinism in the Saprininae subfamily; a dorsal habitus image of a representative of each taxon is included.

## Supplementary Material

XML Treatment for Paramyrmetes

XML Treatment for Paramyrmetes foveipennis
